# Topology of *WFS1* Variants Linked With Islet Function and Higher Risk of Urological Symptoms in *WFS1*-Associated Disease

**DOI:** 10.1155/pedi/9955995

**Published:** 2025-07-31

**Authors:** Juan-juan Zhang, Tong-tong Dai, Jun-qi Wang, Ming-yue Yin, Yuan-yan Yang, Li Jiang, Bei-jun Xia, Zhuo-zhou Cui, Wen-li Lu, Rong-gui Hu, Chuan-yin Li, Zhi-ya Dong, Yuan Xiao

**Affiliations:** ^1^Department of Pediatrics, Ruijin Hospital, Shanghai Jiao-Tong University School of Medicine, No. 197, Ruijin 2nd Road, Shanghai 200025, China; ^2^Department of Colorectal Surgery and Oncology, Key Laboratory of Cancer Prevention and Intervention, China National Ministry of Education, Key Laboratory of Molecular Biology in Medical Sciences, Hangzhou, Zhejiang Province, China; ^3^The Second Affiliated Hospital, Zhejiang University School of Medicine, No. 88, Jiefang Road, Shangcheng District, Hangzhou 310009, Zhejiang, China

**Keywords:** β-cell function, protein structural analysis, variant-phenotype association, *WFS1*-associated disease, Wolfram syndrome type 1 gene (*WFS1*)

## Abstract

Wolfram syndrome type 1 gene (*WFS1*), which encodes a transmembrane (TM) structural protein (wolframin), is essential for several biological processes. Mutations of *WFS1*, autosomal dominant or recessive inherited, are related to a broad clinical spectrum. Molecular genetic tests were performed, and clinical phenotypes of three WFS1-associated cases were evaluated. The expression of *WFS1*, viability, and endoplasmic reticulum (ER) stress of the MIN6 cell and structural analysis of the variant WFS1 protein were revealed. Furthermore, a total of 75 pathogenic *WFS1* variants from ClinVar were included to analyze variant-phenotype association. Genetic testing revealed 3 mutations with unclear pathogenicity in *WFS1* of the 3 patients with early-onset diabetes, including c.613G >A (p.G205S), c.2053C >T (p.R685C), and c.169G >A (p.A57T). Decreased expression, reduced β-cell viability and enhanced ER stress were found in all variants. Protein stability and structural analysis showed increased protein stability and molecule flexibility of variants p.R685C in the ER-lumenal domain and p.A57T in the ATP6VIA-interaction region, while destabilized protein and rigidificated structure by p.G205S variant in the EF-hand domain at the cytoplasm region. Remarkably, topology was found an independent risk factor with urological symptoms (USs) (*p*=0.007, odds ratio [OR] 4.768 [95% confidence interval (CI): 1.531–14.854]). Surprisingly, variants in the cytoplasm had the highest risk with US than ones in the ER-lumenal domain (*p*=0.008, OR 22.013 [95% CI: 2.270–213.428]). The functional analysis of the three variants of uncertain significance in *WFS1* indicated a quantitative and qualitative damage to wolframin with proven pathogenicity. The topology of the WFS1 protein may play an important role in the pathogenesis of β-cell and urological defects in *WFS1*-associated disease.

## 1. Introduction

The human nuclear Wolfram syndrome type 1 gene (*WFS1*) was identified in 1998 [1]. The gene is situated on chromosome 4p16 and comprises eight exons, encoding the WFS1 protein, commonly referred to as wolframin. Wolframin is an endoplasmic reticulum (ER) membrane protein characterized by multiple transmembrane (TM) domains and consists of 890 amino acids [2]. WFS1 is predominantly localized in the mitochondria-associated ER membranes, which function as contact sites between the ER and mitochondria [3]. Accordingly, wolframin was divided by ER membranes into three regions, including one cytoplasmic part, eleven TM parts, and one ER-lumenal domain, while the protein also had one EF-hand domain, C-terminal OB-fold domain, and one disordered region [4]. WFS1 is ubiquitously expressed across various tissues, with the highest expression levels found in pancreatic β-cells, as well as in the heart and brain, particularly within the hippocampus, amygdaloid region, and olfactory tubercle [5].

Wolfram syndrome-1 (WS1) (OMIM 222300) is a rare autosomal recessive multisystem disorder caused by biallelic mutations in *WFS1*, with a prevalence ranging from 1/770,000 to 1/54,478. WS1 is characterized by a constellation of features, including optic atrophy (OA), diabetes mellitus (DM), diabetes insipidus (DI), and sensorineural deafness. Additionally, patients may experience urological symptoms (USs), psychiatric symptoms (PSs), and neurologic symptoms [6]. In 2011, a newly identified disease entity related to WS1 was termed Wolfram-like syndrome (WFLS; OMIM 614296). This syndrome is attributed to heterozygous mutations in the *WFS1* gene and is characterized by the manifestation of one or more symptoms commonly associated with WS1 [7]. Multiple large-scale genome-wide association studies (GWAS) have identified that several single-nucleotide polymorphisms (SNPs) in *WFS1* that are linked to an elevated genetic risk for type 2 DM (T2DM) [8]. The frequency of the WFLS is not well known. However, a study in the UK population showed that the WS1 variant carrier frequency was 1/354 [9].

The prognosis of WS1 is poor, with a rapidly progressive clinical course that results in a premature death at a mean age of 30 years (ranging 25–49 years) [2]. Consequently, elucidating the pathogenesis of WS and WFLS may yield valuable insights into the underlying mechanisms of other forms of diabetes, including T2DM. This study identified four mutations in the *WFS1* gene in three cases with early-onset diabetes from the Chinese Han population. Furthermore, 3 of these mutations, namely c.613G >A (p.G205S), c.2053C >T (p.R685C), and c.169G >A (p.A57T) remain uncertain significance in the ClinVar database. The biochemical properties of *WFS1*, encompassing protein expression and β-cell viability, along with the effects of *WFS1* variants on stability as predicted by structural modeling, were assessed to investigate the functions of the mutations. This assessment was to clarify the pathogenic processes of WS1 or WFLS and to examine the relationship between the functional attributes and clinical symptoms linked to the three mutations.

## 2. Materials and Methods

### 2.1. Patients

Three patients in all, two male and one female, ages 7–14, were included in the research (Table 1). The lineage of these families indicated increased blood glucose levels in at least two generations. According to clinical features and laboratory test findings, diagnoses of type 1 DM (T1DM) or T2DM were excluded using the diagnostic parameters set out by the International Society for Pediatric and Adolescent Diabetes (ISPAD) in 2022 [10]. Genetic testing was performed on the three individuals and their parents to confirm an appropriate diagnosis. This investigation was approved by the Ethics Committee of Ruijin Hospital (2021–261), Shanghai Jiao Tong University, and signed informed permission was acquired from all participants before the study began.

### 2.2. Mutation Screening

Blood samples were obtained from the patients and their progenitors. Genomic DNA was collected with a blood genomic DNA extraction kit, following the manufacturer's instructions (Tiangen Biochemical Science and Technology, Beijing, China). Library preparation and exome capture were executed using the Hieff NGS OnePot DNA Library Prep Kit for Illumina (Yeasen Biotechnology, Shanghai, China) with a capture probe from the Twist Custom Panel (Twist Bioscience, CA, United States). High-throughput sequencing (HTS) of the libraries was conducted in PE100 mode on the DNBSEQ-T7 platform (UWIC). The sequencing data quality was evaluated using FASTQC software (v0.11.8), followed by alignment of the reads to the reference genome (GRCh37/hg19) using BWA software. Mutations were ultimately confirmed using Sanger sequencing (Weyhams Bio; forward primer-ACACGACGCTCTTCCGATCTTGGATGTGCCTGACCTTGAC, reverse primer-CCTTGGCACCCGAGAATTCCACATGTTTGGACGCTGTTGCA). The pathogenicity of the discovered mutations was assessed based on the standards set out by the American College of Medical Genetics and Genomics (ACMG) [11].

### 2.3. Plasmid Construction

The cDNA containing *WFS1*, obtained from Tsingke Biotech Co., Ltd. (Beijing, China), was used as the template and then cloned into the 5′ flag-tagged pCDNA3.0 vectors (VECs). Targeted mutagenesis was applied to alter the *WFS1* gene, utilizing the primers specified in Table S1. Positive clones were chosen and sequenced to confirm the existence of the desired alterations.

### 2.4. Cell Culture and Transfection

The human MIN6 cell line was acquired from Fu Heng Biology (Shanghai, China) and cultured in Dulbecco's modified Eagle's medium (DMEM, Life Technologies) enriched with 10% fetal bovine serum (FBS), 100 U/mL penicillin, 100 mg/mL streptomycin (both from Gibco, Loyola), and 0.5% β-mercaptoethanol (Pricella, Wuhan, China) at 37°C in a humidified environment with 5% CO2. Plasmids were transfected into MIN6 cells using Lipofectamine 8000 (Life Technologies, Carlsbad, CA, United States), in accordance with the manufacturer's guidelines. MIN6 cells were harvested at 48 h post-transfection for subsequent analysis.

### 2.5. Immunoblotting

Cells underwent sodium dodecyl sulfate-polyacrylamide gel electrophoresis (SDS-PAGE) and were then transferred to polyvinylidene fluoride (PVDF) membranes (Bio-Rad). The blots were treated with primary antibodies targeting glyceraldehyde-3-phosphate dehydrogenase (GAPDH; 1:5000, 60,004-1-Ig, Proteintech, China) and Flag (1:1000, 20543-1-AP, Proteintech Group, Chicago, United States). Subsequently, secondary antibodies coupled with horseradish peroxidase (HRP) were used, and the resultant signals were observed using a Tanon 5200 Imaging System (Tanon, Shanghai, China). To assess WFS1 protein stability, MIN6 cells overexpressing wild type (WT) or variant *WFS1* were treated with the protein synthesis inhibitor cycloheximide (CHX; 50 μg/ml, tsbiochem, Cat# T1225, CAS 66-81-9). Cells were harvested at 0, 2, 4, 6, 8, and 10 h post-treatment for Western blot analysis. Subsequent immunoblot examination of Akt phosphorylation was conducted sequentially on the same PVDF membrane, first probing for phosphorylated Akt (Ser473) and then for total Akt. The PVDF membranes were incubated for 8 min in Restore PLUS buffer (Thermo Scientific) and then blocked with 5% BSA between probing stages. GAPDH functioned as a loading control for all immunoblot assays. All Western blot analyses were performed using DMEM with a glucose concentration of 1 g/L. Digital images were acquired with a Bio-Rad ChemiDoc MP and then processed with ImageJ software.

### 2.6. Quantitative Real-Time PCR

MIN6 cells overexpressing WT or variant *WFS1* were exposed to 10 nM thapsigargin (tsbiochem, Targetmol, Cat#TQ0302) for 6 h. Total RNA was extracted from MIN6 cells to perform subsequent qPCR analysis of ER stress markers (*GRP78*, glucose-regulated protein of 78*; ATF6ɑ*, activating transcription factor 6ɑ*; sXBP1*, X-box binding protein 1) using RNeasy Mini Kit (Accurate biology, Cat#AG21022) and reverse-transcribed using cDNA Reverse Transcription Kits (Accurate biology, Cat#AG11728). The qPCR was performed in 3 replicates for each sample (SYBR Green Pro Taq HS qPCR Kit II, Accurate Biology, Cat#AG11719). All the qPCR primer sequences used in this study are listed in Table S2.

### 2.7. Structural Modeling

The AlphaFold3 Protein Structure Database has generated the model structure of WFS1 [12]. The effects of *WFS1* variants on protein stability and their interactions with other proteins were determined and predicted by using the Dynamut and Dynamut2 server (http://biosig.unimelb.edu.au/dynamut/; https://biosig.lab.uq.edu.au/dynamut2/) and PyMOL software (v.3.0.3; PyMOL Molecular Graphics System v. 2.0, Schrödinger Inc., New York, NY, USA).

### 2.8. Bioinformatics Analysis

WFS1 variant data were collected from ClinVar [13], including variant pathogenic classification, exons, domains, topology, the region interacting with V-type proton ATPase catalytic subunit A (ATP6VIA), and variant-related clinical phenotypes.

### 2.9. Statistical Analysis

One-way analysis of variance (ANOVA) was used to ascertain the statistical disparities between groups in immunoblotting data. The Kruskal–Wallis *H* test with multiple test adjustments via the Bonferroni correction was utilized to evaluate the differences in variant classification across groups. Two-way ANOVA was performed to assess differences in protein stability across repeated time measurements and expression of ER stress markers by qPCR among experimental groups. The genotype-phenotype correlation of the *WFS1* gene was analyzed by using binary logistic regression, and Hosmer–Lemesho (H-L) test was used to evaluate the model goodness of fit. A *p* value of less than 0.05 was considered to be statistically significant. Statistical analyses were performed using the IBM SPSS 27.0 (Armonk, New York) and the GraphPad Prism 10.1.2 (San Diego, CA, United States).

## 3. Results

### 3.1. Clinical Characteristics of Cases With *WFS1*-Associated Disease

#### 3.1.1. Case 1

A Chinese girl with presumed diabetes since the age of 13.5, without episodes of diabetic ketoacidosis (DKA) had a history of bipolar disorder and had been taking Seroquel for 1 year. She did not have the typical symptoms such as polydipsia, polyuria, polyphagia, and wasting. Her (maternal) grandmother and mother were both diagnosed with diabetes in middle age. Her mother also had pregnancy-induced hypertension during the delivery, whereas her blood pressure was normal. She was 1.67 m (+1.62 SD) tall, weighed 58.8 kg (+1.44 SD), had a body mass index (BMI) of 21.1 kg/m^2^ (+0.92 SD), and a Tanner III pubertal stage at the first visit. No growth and development delays were found.

**Figure 1:**
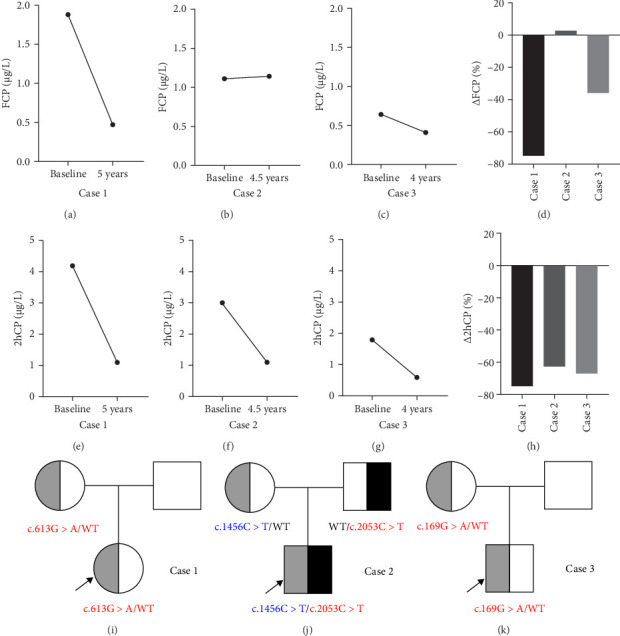
Islet function of *WFS1*-associated disease and pedigree of the cases. (A, E) For Case 1, level of FCP and 2hCP at baseline and 5-year follow-up. (B, F) For Case 2, level of FCP and 2hCP at baseline and 4.5-year follow-up. (C, G) For Case 3, level of FCP and 2hCP at baseline and 4-year follow-up. (D, H) Change rate of FCP (ΔFCP) and 2hCP (Δ2hCP) at last visit compared with data at baseline for 3 cases. (I–K) Squares represent males and circles represent females. Filled black and slash symbols indicate members carrying mutations, and empty symbols indicate family members without mutations. The case is marked with a black arrow. 2hCP, 2-h postprandial C-peptide; FCP, fasting C-peptide.

The results of the Islet autoantibodies, including glutamic acid decarboxylase antibodies (GADAs), islet cell antibodies (ICAs), and insulin autoantibodies (IAAs), were negative. Both the fasting and 2-h postprandial C-peptide (FCP and 2hCP) levels exhibited a marked decline over the course of the 5-year follow-up period, with FCP decreasing from 1.88 μg/L at the onset to 0.47 μg/L. The percentage change in FCP (ΔFCP) was −75.0%, while the percentage change in 2hCP (Δ2hCP) was −73.6% with 4.20 μg/L at the onset to 1.11 μg/L (Figure 1A,D,E,H). Additionally, thyroid nodules were identified, classified as TI-RADS 3. No abnormalities were found in her blood lipid profile, liver and renal function, electrolytes, muscle enzymes, and other hormones, including thyroxine and adrenocortical hormones. The imaging examination, such as echocardiogram and abdominal ultrasound, revealed no abnormalities. She was prescribed multiple daily insulin injections (increasing the daily insulin dosage to 0.79 IU/kg/day at last visit) and oral metformin, but glycemic control remained poor (DKA history during the 5-year follow-up). Her last ophthalmologic, audiogram screening, and urinalysis were normal.

#### 3.1.2. Case 2

Our second case is a boy who was diagnosed with diabetes at the age of 7 at our center. The patient presented with symptoms such as polyphagia and emaciation, but did not display the typical symptoms of polydipsia, polyuria, and admissions for DKA. Besides, His grandfather was also diagnosed with type 2 diabetes, while both his father and paternal aunt had mildly elevated blood glucose levels. His younger brother was healthy by now. At the age of 10 and 3 months, he had growth retardation with a height of 1.30 m (from −0.80 SD at onset to −1.73 SD), but normal BMI. One and a half years later, he attained a height of 1.44 m (−0.98 SD) and was in the normal pubertal stage.

His HbA1c was consistently below 7.5% for several years, requiring only a small dose of insulin therapy (daily insulin dosage: 0.11–0.47 IU/kg/day prior to the onset of puberty, rising to 0.80 IU/kg/day after puberty). Throughout this period, the islet autoantibodies remained negative. The FCP level remained steady 4.5 years later (from 1.11 μg/L at the onset to 1.14 μg/L, ΔFCP: +2.7%), while the 2hCP level declined by 63.0% (from 3.00 μg/L at the onset to 1.11 μg/L) (Figure 1B,D,F,H). No abnormalities were found in his blood lipid levels, liver or renal function, electrolytes, muscle enzymes, and other endocrine hormones. The imaging examinations, including echocardiogram and abdominal ultrasound, yielded normal results. By the age of 10, routine audiogram revealed the presence of subtle sensorineural hearing loss. At the last visit when he was 12 years old, peripheral neuropathy was identified through electromyography. However, no optic nerve atrophy was observed during the ophthalmology examination, nor were there, and clinical features of DI or ataxia. He didn't have diabetes-related complications, such as proteinuria or retinopathy in both eyes, during the follow-ups.

#### 3.1.3. Case 3

A 12-year-old boy was diagnosed with diabetes with typical diabetic symptoms (polyphagia, polydipsia, polyuria, and wasting) and diabetic ketosis (DK), but without acidosis. His history of birth and developmental milestones was unremarkable. A family history of diabetic disease is absent. At the last visit, when he was 15.5 years old, his height was 1.70 m (−0.17 SD), his weight was 52.9 kg (−0.49 SD), and his BMI was 18.3 kg/m^2^ (+0.58 SD). Additionally, he exhibited normal development of sexual characteristics.

A small dose of multiple daily insulin injections (daily insulin dosage: 0.14–0.76 IU/kg/day after puberty) and oral metformin were prescribed, which resulted in improved his glycemic control (HbA1c from 13.4% at the onset to 5.8% at the last visit). The islet autoantibodies were always negative. Both FCP and 2hCP levels decreased over the 4-year follow-up period (FCP from 0.64 μg/L at the onset to 0.41 μg/L, ΔFCP: −35.9%; 2hCP from 1.79 μg/L at the onset to 0.59 μg/L, Δ2hCP: −67.0%, respectively) (Figure 1C,D,G,H). Despite comprehensive physical examinations, biochemical and endocrinological investigations, along with extensive investigations (including an audiogram test, an ophthalmology review, an echocardiogram, and an electromyography), no objective evidence of DI, sensorineural hearing loss, optic nerve atrophy and neuromuscular disorder, or other underlying diabetes-related complications could be identified.

The clinical characteristics of the cases were summarized in Table 1.

### 3.2. Results of Genetic Testing

Based on the features of juvenile-onset diabetes with negative islet autoantibodies and psychiatric disorders, monogenic diabetes was suspected, and consent was obtained for the next-generation sequencing to analyze diabetes related genes in these three pedigrees.

For case 1, a maternally inherited heterozygous missense variant (NM_001145853, c.613G >A, p.G205S) in exon 5 of *WFS1* (Figure 1I) was identified and classified as variant of uncertain significance (VUS) according to the ACMG guideline (PM2 + PP1 + BP6) (Table 2). The variant was also reported as VUS in the ClinVar database.

For case 2, 2 variants (NM_006005) were identified in exon 8 of *WFS1* (Figure 1J): the paternal inherited one was a missense variant (c.2053C >T, p.R685C), the maternal inherited one was a nonsense variant (c.1456C >T, p.Q486X). The former variant is considered likely pathogenic (LP) according to the ACMG guideline (PM1 + PM2 + PM5 + PP1 + PP3) (Table 2). Conflicting interpretations of pathogenicity were reported in ClinVar. The latter variant is classified as pathogenic variant according to the ACMG guideline (PVS1 + PP1 + PP5 + PM2).

For case 3, a maternally inherited heterozygous missense variant NM_006005 (c.169G >A, p.A57T) in exon 2 of *WFS1* (Figure 1K) was identified and classified as VUS according to the ACMG guideline (PM1 + PM2) (Table 2). Conflicting interpretations of pathogenicity were reported in ClinVar.

The *WFS1* gene variants and classification based on the ACMG guidelines are shown in Table 2, and the specific classification reasons can be seen in Tables S3–S5. The pedigree of the cases' families is shown in Figure 1I–K. To investigate the relevant pathogenic mechanisms, we conducted a functional study of the three missense variants in the *WFS1* gene.

### 3.3. Expression of Recombinant *WFS1* Variants and β-Cell Viability In Vitro

**Figure 2:**
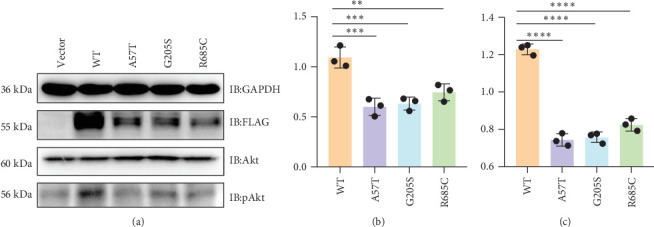
Protein expression levels and cell viability in MIN6 cells. (A) Detection of protein levels in WT *WFS1* and its variants by immunoblotting. Immunoblot analysis of WFS1, p (473)-Akt, and total Akt expression in MIN6 cells transduced with WT *WFS1* and its variants. (B) Quantification of protein levels in MIN6 cells with *WFS1* variants relative to WT control cells. (C) Quantification of p (473)-Akt/Akt in MIN6 cells with *WFS1* variants relative to WT control cells. Data are represented as mean ± SD from three independent experiments. Statistical significance was determined by one-way ANOVA test. *⁣*^*∗∗*^*p*  < 0.01,*⁣*^*∗∗∗*^*p*  < 0.001,*⁣*^*∗∗∗∗*^*p*  < 0.0001.

To investigate the expression of variants and WT *WFS1* in vitro, empty VEC or FLAG-tagged WT *WFS1*/*WFS1* (c.613G >A)/*WFS1* (c.2053C >T)/*WFS1* (c.169G >A) was transfected into MIN6 cells. The protein levels of the FLAG-tagged protein and GAPDH were detected by immunoblotting, and the results showed that the expression level of the variant *WFS1* was markedly lower than that of the WT *WFS1* (Figure 2A,B).

It has been reported that WFS1 confers a functional and a survival advantage to β-cells under ER stress by increasing insulin gene expression and downregulating the Chop-Trib3 axis, thereby activating Akt pathways [14]. The phosphorylation of Akt (Ser473) was found to decrease significantly upon WFS1 variants in MIN6 cells (Figure 2A,C), indicating that the variants may suppress β-cell viability. According to the functional study results, pathogenicity classification by ACMG of the three VUS variants for cases 1–3 were upgraded to LP, P, and LP, respectively (Table 2, by adding PS3, i.e., well-established in vitro or in vivo functional studies supportive of a damaging effect on the gene or gene product).

However, no notable differences were observed among the three variants with regard to protein expression or Akt phosphorylation levels. Nevertheless, a discernible trend emerged, whereby the A57T variant exhibited the most pronounced impact on function, while the R685C variant demonstrated the least significant effect.

### 3.4. Impact of *WFS1* Variants on Protein Stability and ER Stress in β-Cells In Vitro

**Figure 3:**
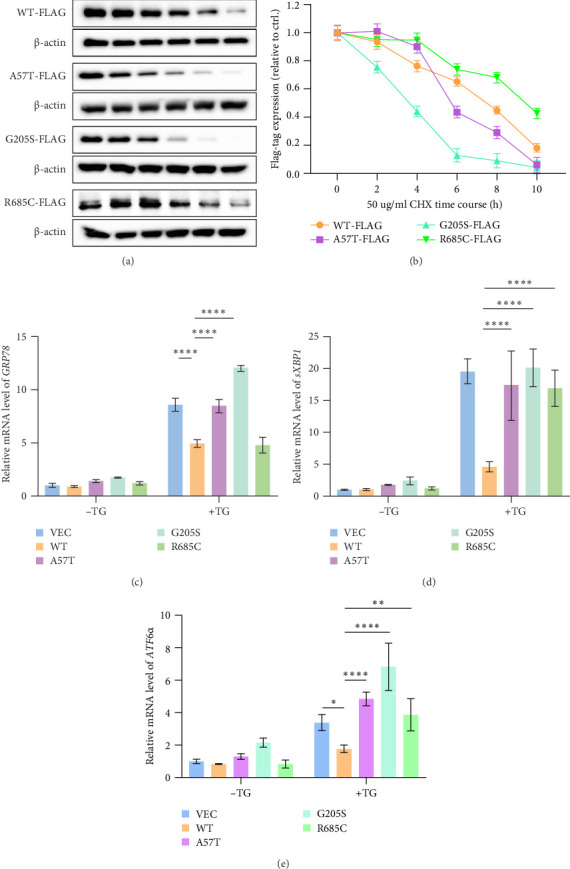
Protein expression levels and cell viability in MIN6 cells. (A) Detection of protein levels in WT *WFS1* and its variants by immunoblotting. Immunoblot analysis of WFS1 in MIN6 cells overexpressing WT or variant *WFS1* treated with the protein synthesis inhibitor cycloheximide (CHX; 50 μg/ml) at 0, 2, 4, 6, 8, and 10 h posttreatment. (B) Quantification of protein levels in MIN6 cells with WFS1 variants relative to WT control cells across time. (C–E) Quantification of ER stress markers by qPCR analysis (*GRP78*, glucose-regulated protein of 78; *ATF6ɑ*, activating transcription factor 6ɑ; *sXBP1*, X-box binding protein 1) in MIN6 cells with *WFS1* variants relative to empty vector (VEC) and WT control cells, followed by 10 nM thapsigargin stimulation for 6 h. Data are represented as mean ± SD from four independent experiments. Statistical significance was determined by two-way ANOVA test. *⁣*^*∗*^*p*  < 0.05, *⁣*^*∗∗*^*p*  < 0.01, *⁣*^*∗∗∗∗*^*p*  < 0.0001.

Both WT and variant *WFS1* protein levels decreased significantly over time (*p*  < 0.0001) (Figure 3A,B). Among the variants, G205S exhibited the poorest stability. Notably, A57T and R685C showed protein levels comparable to or higher than WT at 4 h (R685C vs. WT at 4 h: *p*=0.0265). However, A57T levels dropped sharply after 6 h (A57T vs. WT at 6 h: *p*=0.0074), while R685C consistently maintained higher protein levels than all other groups throughout the time course (R685C vs. WT at 10 h: *p*=0.0036), indicating superior stability.

WT *WFS1* significantly suppressed ER stress induction (vs. VEC control) (Figure 3C–E). Both A57T and G205S variants exhibited severely compromised suppression versus WT (all markers: *p*  < 0.0001), with G205S showing the most profound functional impairment. R685C demonstrated the mildest impairment, with near-preserved suppression of *GRP78* expression comparable to WT (Figure 3C). The R685C variant exhibited significantly lower *GRP78* expression than both the G205S and A57T variants (*p* < 0.0001) (Figure 3C). While *sXBP1* levels showed no significant differences among the three variants (Figure 3D), *ATF6α* expression in R685C was lower compared to G205S (*p*  < 0.0001) but remained comparable to A57T (Figure 3E).

### 3.5. 3D Modeling and Structural Analysis

**Figure 4:**
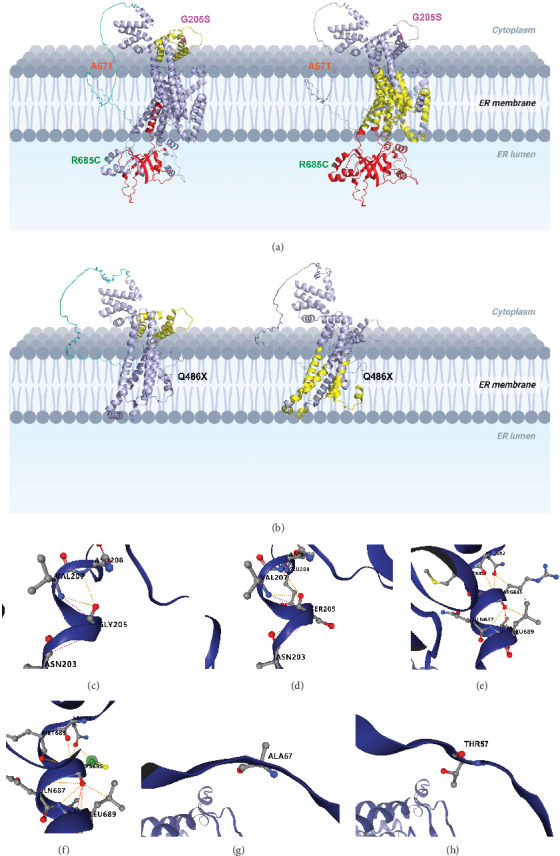
3D modeling and structural analysis of *WFS1* variants. (A) Wild-type WFS1 3D model generated from Alphafold3. Domains (left): disordered region (cyan), EF-hand domain (yellow), C-terminal OB-fold domain (red). Topology regions (right): cytoplasmic region (purple), TM regions (yellow), ER lumenal domain (red). Variant locations are marked by colors: p.G205S (pink), p.R685C (green), A57T (red). Created in BioRender. Zhang, J. (2025) https://BioRender.com/o51m225. (B) WFS1 p.Q486X. Domains (left): disordered region (cyan), EF-hand domain (yellow). Topology regions (right): cytoplasmic region (purple), TM regions (yellow). Nearly half of the length of the protein is truncated, including the C-terminal OB-fold domain, TM regions 6–11, and the ER lumenal domain. Conformational changes of WFS1 (p.Q486X) variant were observed. Created in BioRender. Zhang, J. (2025) https://BioRender.com/x75r125. (C, D) Interactomic interactions analysis of G205S variant. Wild-type residues (C) and variant residues (D). A new hydrogen bond (red dashes) between the amino acids SER205 and GLU209 in the variant. (E, F) Interactomic interactions analysis of R685C variant. Wild-type residues (E) and variant residues (F). Loss of a hydrophobic bond (green dashes) between the 685 residue and LEU689. (G, H) Interactomic interactions analysis of A57T variant. Wild-type residues (G) and variant residues (H). A new hydrogen atom in the A57T variant (red balls).

The topology structure indicated that all three variants were not located in the TM domains. The G205S and A57T variants were suited in the cytoplasm region, while the R685C variant was located in the ER lumenal domain. Moreover, according to the function part, the G205S variant lay in the EF-hand domain, and A57T was in the disordered region as well as the region that interacted with ATP6VIA. Notably, the R685C variant, derived from case 2, was not located in any functional domain (Figure 4A). In contrast, the other pathogenic variant (p.Q486X) resulted in the truncation in the C-terminal OB-fold domain, TM regions 6–11, and the ER lumenal domain, leading to a severe compromise in protein structure stability (Figure 4B).

Subsequently, DynaMut was employed to predict the regional protein stability and molecule flexibility, demonstrating increased protein stability and molecule flexibility by R685C and A57T variants while destabilizing protein and rigidification of the structure by G205S variant (Table 3). Interactomic interactions analysis by DynaMut2 demonstrated that the variants might disrupt the interactions between the WFS1 protein amino acids to different extents. The G205S variant may lead to the increase of a hydrogen bond between the amino acids SER205 and GLU209, while the R685C variant may cause loss of a hydrophobic bond between the 685 residue and LEU689. A new hydrogen atom may appear in the A57T variant.

### 3.6. Function Annotations and Variant-Pathogenicity Correlation of *WFS1*

**Figure 5:**
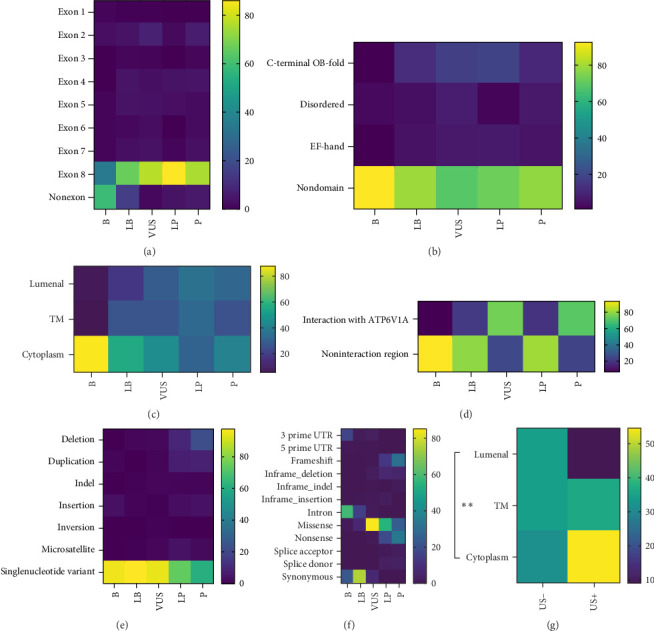
Variant-pathogenicity and variant-phenotype correlation of *WFS1*. (A–F) Correlation of variant pathogenic classification with exons (A), domains (B), topology (C), ATP6VIA-interaction regions (D), variant types (E), and molecular consequence (F). Correlation between topology of variants and US (G). Data represented the distribution of each group (each square in the heatmap), and statistical significance was determined by Kruskal–Wallis *H* test (A–F) and binary logistic regression (G). ATP6VIA, V-type proton ATPase catalytic subunit A. SNV, single-nucleotide variant; TM, transmembrane parts; US, urological symptom.*⁣*^*∗∗*^*p*  < 0.01.

A total of 1732 variants in *WFS1* have been reported by ClinVar up to now. To figure out whether the locations of mutation on the protein structure are associated with the pathogenicity of these variants, a correlation was established between the pathogenic classification of the variants and the following: exons, domains, topology, and the ATP6VIA-interaction region. With regard to exons, it was found that exons 2–8 exhibited a greater degree of pathogenicity in comparison to nonexon variants. However, no discernible differences were observed among exons. Nevertheless, the distribution of variants was found to be highest in exon 8 (Figure 5A). For domains, variants in the C-terminal OB-fold domain exhibited greater pathogenicity than nondomain variants (*p*=0.002). In contrast, no differences in pathogenicity were observed between EF-hand domains and other domains or non-domains (Figure 5B). With regard to topology, both variants in the ER-lumenal domain and TM had higher risk than those in the cytoplasmic part (both *p*  < 0.001) (Figure 5C). The pathogenicity of variants in the ATP6VIA-interaction region was found to be higher than that of noninteraction regions (*p*=0.002) (Figure 5D). With regard to variant types, single-nucleotide variants (SNVs) were observed to be less pathogenic than deletion, duplication and microsatellite variants (*p*  < 0.001, *p*  < 0.001 and *p*=0.009, respectively). Moreover, insertion variants were found to be less harmful than deletion and duplication variants (*p*=0.037 and *p*=0.03, respectively) (Figure 5E). With regard to the molecular consequence, intron variants were observed to have a lesser impact than other groups (all *p*  < 0.01) with the exception of 5′ prime untranslated region (5′-UTR) variants. Synonymous variants were also found to be more benign than other groups (all *p*  < 0.001), with exception of 3′ prime UTR (3′-UTR) variants and inframe_indel variants. Mutations in the 3′-UTR were observed to have a less detrimental effect than those with missense, in-frame deletion, nonsense, frameshift, splice donor (all *p*  < 0.01), and splice acceptor (*p*=0.005) mutations. Furthermore, 5′-UTR variants demonstrated a reduced risk compared to nonsense (*p*=0.002), frameshift (*p*=0.002), and splice donor variants (*p*=0.048). The results demonstrated that variants with nonsense and frameshift were more pathogenic than missense variants (both *p*  < 0.001) and in-frame insertion variants (both *p*=0.038) (Figure 5F).

### 3.7. Pathogenic Variant-Phenotype Correlation of *WFS1*

To investigate the correlation between the pathogenic *WFS1* variants and clinical phenotypes, 75 variants classified as pathogenic from the ClinVar library (75 out of 93 pathogenic variants had recorded clinical phenotypes) were analyzed for the influence of clinical manifestations, such as DM, OA, DI, hearing impairment (HI), endocrine symptom (ES) other than DM, neurological symptoms (NSs), USs, PSs by covariates, including exons, domains, topology, ATP6VIA-interaction region, variant types and molecular consequence. Topology was found to be an independent risk factor for US (*p*=0.007, odds ratio [OR] 4.768 [95% confidence interval (CI): 1.531–14.854], *p* value for H-L test > 0.05, indicating a good model fit). Surprisingly, variants in the cytoplasm had the highest risk of US, being almost 22 times more likely to suffer from US than those in the ER-lumenal domain (*p*=0.008, OR 22.013 [95% CI: 2.270–213.428]), while no significant differences were found between TM and the ER-lumenal domain (Figure 5G).

In addition to variant location and type, the effect of protein structure modification on the clinical phenotypes was then analyzed with the predicted stability change (ΔΔ*G*^Stability^) of 18 missense variants from the above 75 variants, and no significant association with the incidence of each manifestation was found.

## 4. Discussion

Predicting prognosis based on *WFS1* genotype will enhance patient treatment and counseling. Nonetheless, prior investigations and a recent comprehensive literature review on the clinical manifestation of WS1 indicate that the pathogenic variation does not forecast the phenotype [15–18], and no common founder mutation or mutation hotspots have been found in the WFS1 gene [19].

Notably, the functional studies of the VUS variants in three cases upgraded the pathogenicity classification by ACMG to LP or P. In our study, cases 1 and 3 were diagnosed with WFLS with heterozygous missense mutations (both LP), and case 2 was diagnosed with WS1 with compound heterozygous mutations (one missense and one nonsense mutation for P). For DM, among the three patients, case 1 had the worst glycaemic control and loss of islet function despite being the last to be diagnosed with diabetes, while case 2 had the most stable islet function and good glycaemic control, but with the earliest onset time of diabetes. And case 1 also had early-onset of PS, whereas case 3 had only DM to date, and case 2 had already suffered 4 WS1-related symptoms. The findings were in accordance with the previous study, which indicated that within the spectrum of WFS1-associated disorders, or “wolframinopathies”, WFLS caused by a single allele mutation in the *WFS1* gene had a milder clinical presentation than WS1 with double allele mutations [20].

However, in vitro experiments, the β-cell viability seemed to be less damaged by the *WFS1* mutation (G205S) from case 1 than by the mutation (A57T) from case 3. The finding was also consistent with the expression of the WFS1 protein, though it lacked statistical significance. However, these findings contrast with the protein stability and ER stress responses observed in MIN6 cells expressing *WFS1* variants. This suggests that disease progression in β-cells depends more critically on the functional integrity of WFS1, particularly its ability to regulate ER stress through mechanisms such as GRP78 suppression-than on mere expression levels. This is exemplified by the R685C variant, which maintains near-normal *GRP78* inhibitory activity despite reduced protein expression. In fact, *WFS1* mutations do not affect mitochondrial function or morphology [21]. It seems to be facilitated by the interactions between the inositol 1,4,5-trisphosphate receptor (IP3R), voltage-dependent anion channel 1 (VDAC1), and glucose-regulated protein 75 (GRP75) [22], as well as neuronal calcium sensor 1 (NCS1) [23] and TOMM20 [24]. These results indicate a broader influence on vesicular transport and the relationship between the ER and mitochondria. The purported effects of the mutations did not correspond with the actual expression of the WFS1 protein [25]. This suggests that although the inactivation of the WFS1 protein is essential for WS, the mechanism underlying this inactivation is not well understood [21].

Moreover, the variant of case 1 located in the EF-hand domain at the cytoplasmic region was estimated to have a destabilized effect on the protein and to rigidify the protein structure through structural analysis, which was distinct from other two cases, consistent with the findings in the ER stress. Notably, although the A57T variant exhibited the highest molecular flexibility among *WFS1* variants-exceeding that of R685C-its stabilizing effect was transient. This may be attributed to A57T's localization within a disordered region, where molecular flexibility primarily facilitates conformational equilibrium rather than sustained stability. It has been previously mentioned that folding defects could be a common feature of WFS1 missense mutations [26], indicating that such changes may potentially affect protein folding. Numerous functional investigations have proven the structural instability induced by variations in the ER-lumenal region. It is probable that wolframin will undergo misfolding as a consequence of WFS1 variants in the ER-lumenal domain, leading to a reduction in its half-life and rapid degradation [7]. In individuals with autosomal dominant low-frequency sensorineural hearing impairment (LFSNHI) resulting from heterozygous WFS1 mutations, these structural and molecular events may elevate ER stress and disrupt calcium homeostasis, ultimately resulting in hearing degradation [27]. To the best of our knowledge, there is no published report of wolframin instability outside the ER-lumenal domain and TM regions. However, it has been indicated that misfolding outside of the TM helix of intrinsically disordered proteins (IDPs) may have a greater impact on protein function [28].

Nevertheless, the potential effects of the increased protein stability and molecular flexibility of wolframin (in cases 2 and 3) on protein function remain unclear. A novel *WFS1* c.2421C >G (p.Ser807Arg) mutation was identified in a Chinese family with ADNSHL with an increase in protein stability [29]. The inconsistency of wolframin's overstability and decreased protein expression may be due to protein stability is controlled by post-translational modifications (PTMs). PTMs may occur on particular amino acids located inside regulatory domains of target proteins, referred known as degrons, which serve as signals to accelerate protein degradation (PTM-activated degrons) or to inhibit degradation and stabilize a protein (PTM-inactivated degrons) [30]. A further elucidation of degrons on *WFS1* will be a prospective direction to study the *WFS1* variant's pathogenicity.

For case 2, the onset of HI and NS occurred at an earlier age than the average age of onset reported in the literature (12.5 years for HI and 16 years for NS, respectively) [2]. The loss of the hydrogen bond between the p.C685 and p.L689 residues in the ER-lumenal domain may explain this phenomenon, since the p.G736D variation in the same domain modifies hydrogen bonds and is linked to the deterioration of the helical structure [31]. The p.A684V variation, an additional WFS1 mutation associated with ADNSHL, is anticipated to impair helix A and perhaps disturb the connection between WFS1 and NCS1, since helix A may facilitate intra-ER communication related to NCS1 contacts [27]. The heterozygous missense mutations p.E809K and p.E830A in the ER-lumenal domain, identified in patients with diabetes diagnosed before 12 months and sensorineural deafness diagnosed shortly after birth, have been shown to modify polarity and hydrophobicity, significantly affecting the surface properties and solvent accessibility of wolframin. Vitro studies showed that these variants tended to aggregate and induced robust ER stress [26]. Additionally, the p.Q486X variant resulted in the truncation of the C-terminal OB-fold domain, TM regions 6–11, and the ER lumenal domain. This may potentially impair membrane localization and C-terminal signal transduction. Notably, ADNSHL-related *WFS1* missense variants were predominantly concentrated in the ER-lumenal domain [32]. This may be attributed to the fact that the ER-localized Na^+^/K^+^ATPase beta-1 subunit (ATP1B1) binds to the ER-luminal domain of wolframin [33].

Previously, p.Q486X was reported in two patients with more severe WS1 (with a frameshift variant) [34] and isolated inherited optic neuropathies (IONs) [35]. However, case 2 in our study has not yet exhibited symptoms of OA. The reason may be due to the presence of another missense mutation in our case, compared with other patients with p.Q486X. Biallelic loss-of-function mutations are linked to WS, characterized by insulin-dependent diabetes and OA, with a sensitivity of 79% (95% CI: 75%–83%) and a specificity of 92% (83%–97%) [36]. Furthermore, the onset age for both DM and OA was considerably earlier in individuals with anticipated full loss-of-function mutations than in those with expected partial loss-of-function mutations [37]. Also, DM with OA manifested much sooner in individuals with two nonsense/frameshift genes in contrast to those with none or one nonsense/frameshift variation [38].

Despite the absence of a clear genotype-phenotype association, the previous correlation analysis mainly focused on the functional alterations of wolframin. However, there has been a paucity of discussion on the inheritance pattern of *WFS1* mutations, and the type of mutation [20, 26, 36, 37, 39–47], as well as on phenotype analysis from the perspective of protein structure. Our results indicated that variants located in the C-terminal OB-fold domain, the ER-lumenal domain, TM regions, and the ATP6VIA-interaction region had higher likelihood of pathogenicity. This finding is consistent with the established role of WFS1 in ER stress. Although no significant association with the frequent clinical features, such as DM, OA, HI, and DI, the topology was identified as an independent risk factor for development of US. Interestingly, the variants in the cytoplasm got the highest risk of US, with a prevalence nearly 22 times higher than those variants in the ER-lumenal domain. No significant differences were found between the TM and the ER-lumenal domain. The prevalence of US was determined to be 19.39% in WS1 patients, with neurogenic bladder and upper urinary tract dilatation being the main urological abnormalities. The average age of onset is 20 years, with three prominent peaks: one at 13 years, another at 21 years, and the last at 33 years [2]. There was also an interesting finding that patients with the expression of a defective WFS1 protein have onset of US at 15 years of age, which was earlier than those with no WFS1 protein produced due to mRNA and protein degradation at 30 years of age [41]. This suggests that the role of WFS1 in the mechanism of “uncommon clinical manifestation” may be unique compared with those “common features”. Riachi et al. [48] proposed that mechanisms such as nonsense-mediated decay, methylation signatures, or post-transcriptional controls may influence penetrance.

## 5. Conclusions

In conclusion, the immunoblotting and structure assessment demonstrated that all three mutations impact the function of the WFS1 protein by a decrease in protein expression and a change in protein structure, which subsequently resulted in a reduction in β-cell viability and enhanced ER stress. These findings suggested the pathogenicity of three VUS variants with quantitative and qualitative damage to wolframin. It highlighted the importance of conducting functional analysis to assess the pathogenicity of *WFS1* variants, rather than relying on ACMG prediction alone. It is noteworthy that topology was identified as an independent risk factor for US. Patients with variants in the cytoplasm exhibited a 22-fold increased likelihood of developing US, indicating that the topology of wolframin may play an important role in β-cell and urological defects in *WFS1*-related disease.

## Figures and Tables

**Table 1:** Clinical characteristics of cases with *WFS1*-related disease.

Clinical characteristics	Case 1	Case 2	Case 3
Age at diagnosis (years)	13.5	7	12
Gender	Female	Male	Male
Height SDS at onset	1.62	−0.80	0.53
Weight SDS at onset	1.44	−0.55	−0.04
BMI SDS at onset	0.92	−0.38	−0.32
DM family history	Yes	Yes	No
Disease manifestation			
DM	✔	✔First	✔First
HI	—	✔ (10 years)	—
OA	—	—	—
DI	—	—	—
NS	—	✔ (12 years)	—
PS	✔First (12 years)	—	—
ES	—	✔ (10 years)	—
US	—	—	—
Insulin therapy	Increased dosage	Small dosage	Small dosage
Glycemic control	Poor	Good	Good

*Note:* Blank indicates no onset of clinical manifestations. ES, endocrine symptom other than DM.

Abbreviations: DI, diabetes insipidus; DM, diabetes mellitus; HI, hearing impairment; NS, neurological symptom; OA, optic atrophy; PS, psychiatric symptom; SDS, standard deviation score; US, urological symptom.

**Table 2:** The uncertain clinical significance variants of the patients in this study.

Number	Exon	cDNA	Protein	Genotype	Origin	ACMG by functional studies	
						Before	After
1	5	c.613G >A	p.G205S	Heterozygous	Mother	VUS	LP
2	8	c.2053C >T	p.R685C	Heterozygous	Father	LP	P
3	2	c.169G >A	p.A57T	Heterozygous	Mother	VUS	LP

Abbreviations: LP, likely pathogenic; P, pathogenic; VUS, variant of uncertain significance.

**Table 3:** Wolframin protein stability and molecule flexibility affected by variants.

Protein stability/molecule flexibility	P1 (G205S)	P2 (R685C)	P3 (A57T)
ΔΔ*G*^Stability^	−0.35 kcal/mol	0.24 kcal/mol	0.17 kcal/mol
Predicted stability change	Destabilizing	Stabilizing	Stabilizing
ΔΔ*S*_Vib_ ENCoM	−0.045 kcal.mol^−1^.K^−1^	0.115 kcal.mol^−1^.K^−1^	4.182 kcal.mol^−1^.K^−1^
Δ Vibrational entropy energy	Molecule flexibility↓	Molecule flexibility↑	Molecule flexibility↑

## Data Availability

The data that support the findings of this study are available from the corresponding author upon reasonable request.

## Supporting Information

Supporting Information Table S1. Sequences of the primers used to construct the plasmids in this study. Table S2. The qPCR primer sequences used in this study. Table S3. ACMG classification of Case 1 (c.613G>A, p.G205S). Table S4. ACMG classification of Case 2 (c.2053C>T, p.R685C). Table S5. ACMG classification of Case 3 (c.169G>A, p.A57T).
