# Supplementary material for: Topology of WFS1 Variants Linked With Islet Function and Higher Risk of Urological Symptoms in WFS1-Associated Disease
Source: Pediatr Diabetes. 2025 Jul 31;2025:9955995. doi: 10.1155/pedi/9955995 (PMC12331406; doi:10.1155/pedi/9955995)
Supplement: Supporting Information — Table S1. Sequences of the primers used to construct the plasmids in this study. Table S2. The qPCR primer sequences used in this study. Table S3. ACMG classification of Case 1 (c.613G>A, p.G205S). Table S4. ACMG classification of Case 2 (c.2053C>T, p.R685C). Table S5. ACMG classification of Case 3 (c.169G>A, p.A57T). [file 9955995.f1.docx]

**Supplementary Material**

**Supplementary Table 1.** **Sequences of the primers used to construct the plasmids in this study.**

| **Gene** | **Forward/ Reverse** | **Sequence（5’→3’）** |
| --- | --- | --- |
| *WFS1*(G205S) | Forward | CTGGAGAATGTCAGCCAGGTCAACGAGC |
| *WFS1*(G205S) | Reverse | GACCTGGCTGACATTCTCCAGCAGCTCCGC |
| *WFS1*(R685C) | Forward | CAACATGGCGTGCACCCAGATCCTCTGC |
| *WFS1*(R685C) | Reverse | ATCTGGGTGCACGCCATGTTGGTCTCCTT |
| *WFS1*(A57T) | Forward | CTGGTGTTAGAGACACAGCGGCCCCCGCTGAACCC |
| *WFS1*(A57T) | Reverse | CGGGGGCCGCTGTGTCTCTAACACCAGGGCCA |

**Supplementary Table 2. The qPCR primer sequences used in this study.**

| **RefSeq** | **Gene name** | **Forward Primer Sequence** | **Reverse Primer Sequence** | **Amplicon size** |
| --- | --- | --- | --- | --- |
| NM_022310 | *GRP78* | CTGGGTACATTTGATCTGACTGG | GCTTTTCCTGTTGCTCCATAG | 150bp |
| NM_001271730 | *sXBP1* | GAACCAGGAGTTAAGAACACG | AGGCAACAGTGTCAGAGTCC | 199bp |
| NM_001287231 | *ATF6α* | CAGACTGGTTTCGACAGGTG | CTTCCAGGGAGACACCAGTT | 120bp |
| NM_012583 | *HPRT* | TCAGTCAACGGGGGACATAAA | GGGGCTGTACTGCTTAACCAG | 101bp |

**Supplementary Table 3. ACMG classification of case 1 (c.613G>A, p.G205S).**

| **Classification** | **Explains** |
| --- | --- |
| PM2 | GnomAD genomes homozygous allele count = 0 is less than 2 for AD/AR gene *WFS1*, good gnomAD genomes coverage = 31.6.  GnomAD exomes homozygous allele count = 0 is less than 2 for AD/AR gene *WFS1*, good gnomAD exomes coverage = 61.5. |
| PP1 | Cosegregation with phenotype in multiple affected family members in gene *WFS1* which is associated with Wolfram-Like Syndrome, Wolfram-Like Syndrome, Autosomal Dominant, Wolfram-Like Syndrome, Autosomal Dominant, 614296 and Wolfram-Like Syndrome, Autosomal Dominant, Omim:614296, according to ClinGen Disease Validity, GenCC, Mondo, OMIM, PanelApp and gene2phenotype. |
| BP6 | Combined evidence strength is Supporting (score = 1).  Supporting: LOVD classifies this variant as Likely Benign. |

| **Classification** | **Explains** |
| --- | --- |
| PM1 | Hot-spot of length 17 amino-acids has 30 missense/in-frame variants (12 pathogenic variants, 18 uncertain variants and no benign), which qualifies as moderate pathogenic. |
| PM2 | GnomAD genomes homozygous allele count = 0 is less than 2 for AD/AR gene *WFS1*, good gnomAD genomes coverage = 33.8.  GnomAD exomes homozygous allele count = 0 is less than 2 for AD/AR gene *WFS1*, good gnomAD exomes coverage = 102.5. |
| PM5 | Alternative variant [chr4:6301849 G⇒C](https://varsome.com/variant/hg38/chr4:6301849%20G-C?&annotation-mode=germline) (Arg685Pro) is classified Pathogenic by UniProt Variants (confirmed using the germline classifier). |
| PP1 | Cosegregation with phenotype in multiple affected family members in gene *WFS1* which is associated with Wolfram Syndrome, Wolfram Syndrome 1, Wolfram Syndrome 1, 222300, Wolfram Syndrome 1, Omim:222300, Wolfram Syndrome, 222300 and Wolfram Syndrome, Omim:222300, according to CGD, ClinGen Disease Validity, GenCC, Mondo, OMIM and 2 more. |
| PP3 | MetaRNN = 0.838 is between 0.748 and 0.841 ⇒ supporting pathogenic. |

**Supplementary Table 4. ACMG classification of case 2 (c.2053C>T, p.R685C).**

**Supplementary Table 5. ACMG classification of case 3 (c.169G>A, p.A57T).**

| **Classification** | **Explains** |
| --- | --- |
| PM1 | UniProt protein WFS1_HUMAN region of interest 'Interaction with ATP6V1A' has 263 missense/in-frame variants (38 pathogenic variants, 216 uncertain variants and 9 benign variants), which qualifies as supporting pathogenic. |
| PM2 | GnomAD genomes homozygous allele count = 0 is less than 2 for AD/AR gene *WFS1*, good gnomAD genomes coverage = 34.2.  GnomAD exomes homozygous allele count = 0 is less than 2 for AD/AR gene *WFS1*, good gnomAD exomes coverage = 54.7. |
